# Fredholmness of multiplication of a weighted composition operator with its adjoint on $H^{2}$ and $A_{\alpha}^{2}$

**DOI:** 10.1186/s13660-018-1615-0

**Published:** 2018-01-22

**Authors:** Mahmood Haji Shaabani

**Affiliations:** Department of Mathematics, Shiraz University of Technology, Shiraz, Iran

**Keywords:** 47B33, 47A53, Hardy space, weighted Bergman spaces, weighted composition operator, Fredholm operator

## Abstract

In this paper, we obtain that $C_{\psi,\varphi}^{\ast}$ is bounded below on $H^{2}$ or $A_{\alpha}^{2}$ if and only if $C_{\psi,\varphi }$ is invertible. Moreover, we investigate the Fredholm operators $C_{\psi_{1},\varphi_{1}}C_{\psi_{2},\varphi_{2}}^{\ast}$ and $C_{\psi_{1},\varphi_{1}}^{\ast}C_{\psi_{2},\varphi_{2}}$ on $H^{2}$ and $A_{\alpha}^{2}$.

## Introduction

Let $\mathbb{D}$ denote the open unit disk in the complex plane. The Hardy space, denoted $H^{2}(\mathbb{D})=H^{2}$, is the set of all analytic functions *f* on $\mathbb{D}$ satisfying the norm condition

$$\|f\|_{1}^{2}=\lim_{r \rightarrow1} \int_{0}^{2\pi} \bigl\vert f\bigl(re^{i\theta } \bigr) \bigr\vert ^{2}\frac{d\theta}{2\pi}< \infty. $$

The space $H^{\infty}(\mathbb{D})=H^{\infty}$ consists of all analytic and bounded functions on $\mathbb{D}$ with supremum norm $\| f\|_{\infty}=\sup_{z \in\mathbb{D}}|f(z)|$.

For $\alpha> -1$, the weighted Bergman space $A^{2}_{\alpha}(\mathbb {D})=A^{2}_{\alpha}$ is the set of functions *f* analytic in $\mathbb {D}$ with

$$\|f\|_{\alpha+2}^{2}=(\alpha+1) \int_{\mathbb {D}} \bigl\vert f(z) \bigr\vert ^{2} \bigl(1-|z|^{2}\bigr)^{\alpha}\, dA(z) < \infty, $$

where *dA* is the normalized area measure in $\mathbb{D}$. The case where $\alpha=0$ is known as the (unweighted) Bergman space, often simply denoted by $A^{2}$.

Let *φ* be an analytic map from the open unit disk $\mathbb{D}$ into itself. The operator that takes the analytic map *f* to $f \circ \varphi$ is a composition operator and is denoted by $C_{\varphi}$. A natural generalization of a composition operator is an operator that takes *f* to $\psi\cdot f \circ\varphi$, where *ψ* is a fixed analytic map on $\mathbb{D}$. This operator is aptly named a weighted composition operator and is usually denoted by $C_{\psi,\varphi}$. More precisely, if *z* is in the unit disk, then $(C_{\psi,\varphi}f)(z)=\psi(z)f(\varphi(z))$. For some results on weighted composition and related operators on the weighted Bergman and Hardy spaces, see, for example, [1–14].

If *ψ* is a bounded analytic function on the open unit disk, then the multiplication operator $M_{\psi}$ defined by $M_{\psi }(f)(z)=\psi(z)f(z)$ is a bounded operator on $H^{2}$ and $A_{\alpha }^{2}$ and $\|M_{\psi}(f)\|_{\gamma} \leq\|\psi\|_{\infty}\|f\| _{\gamma}$ when $\gamma=1$ for $H^{2}$ and $\gamma=\alpha+2$ for $A_{\alpha}^{2}$. Let *P* denote the orthogonal projection of $L^{2}(\partial\mathbb{D})$ onto $H^{2}$. For each $b \in{L^{\infty}(\partial\mathbb{D})}$, the Toeplitz operator $T_{b}$ acts on $H^{2}$ by $T_{b}(f)=P(bf)$. Also suppose that $P_{\alpha}$ is the orthogonal projection of $L^{2}({\mathbb{D}}, dA_{\alpha})$ onto $A^{2}_{\alpha}$. For each function $w \in L^{\infty}(\mathbb{D})$, the Toeplitz operator $T_{w}$ on $A^{2}_{\alpha}$ is defined by $T_{w}(f)=P_{\alpha}(wf)$. Since *P* and $P_{\alpha}$ are bounded on $H^{2}$ and $A^{2}_{\alpha }$, respectively, the Toeplitz operators are bounded.

Let $w \in\mathbb{D}$, and let *H* be a Hilbert space of analytic functions on $\mathbb{D}$. Let $e_{w}$ be the point evaluation at *w*, that is, $e_{w}(f)=f(w)$ for $f \in H$. If $e_{w}$ is a bounded linear functional on *H*, then the Riesz representation theorem implies that there is a function (usually denoted $K_{w}$) in *H* that induces this linear functional, that is, $e_{w}(f)=\langle f,K_{w}\rangle$. In this case, the functions $K_{w}$ are called the reproducing kernels, and the functional Hilbert space is also called a reproducing kernel Hilbert space. Both the weighted Bergman spaces and the Hardy space are reproducing kernel Hilbert spaces, where the reproducing kernel for evaluation at *w* is given by $K_{w}(z)=(1-\overline{w}z)^{-\gamma}$ for $z,w \in\mathbb{D}$, with $\gamma=1$ for $H^{2}$ and $\gamma=\alpha+2$ for $A_{\alpha }^{2}$. Let $k_{w}$ denote the normalized reproducing kernel given by $k_{w}(z)=K_{w}(z)/\|K_{w}\|_{\gamma}$, where $\|K_{w}\|_{\gamma}^{2}=(1-|w|^{2})^{-\gamma}$.

Suppose that *H* and $H'$ are Hilbert spaces and $A:H \rightarrow H'$ is a bounded operator. The operator *A* is said to be left semi-Fredholm if there are a bounded operator $B:H'\rightarrow H$ and a compact operator *K* on *H* such that $BA=I+K$. Analogously, *A* is right semi-Fredholm if there are a bounded operator $B':H'\rightarrow H$ and a compact operator $K'$ on $H'$ such that $AB'=I+K'$. An operator *A* is said to be Fredholm if it is both left and right semi-Fredholm. It is not hard to see that *A* is left semi-Fredholm if and only if $A^{\ast}$ is right semi-Fredholm. Hence *A* is Fredholm if and only if $A^{\ast}$ is Fredholm. Note that an invertible operator is Fredholm. By using the definition of a Fredholm operator it is not hard to see that if the operators *A* and *B* are Fredholm on a Hilbert space *H*, then *AB* is also Fredholm on *H*. The Fredholm composition operators on $H^{2}$ were first identified by Cima et al. [15] and later by a different and more general method by Bourdon [2]. Cima et al. [15] proved that only the invertible composition operators on $H^{2}$ are Fredholm. Moreover, MacCluer [16] characterized Fredholm composition operators on a variety of Hilbert spaces of analytic functions in both one and several variables. Recently, Fredholm composition operators on various spaces of analytic functions have been studied (see [13] and [14]).

The automorphisms of $\mathbb{D}$, that is, the one-to-one analytic maps of the disk onto itself, are just the functions $\varphi(z)=\lambda\frac{a-z}{1-\overline{a}z}$ with $|\lambda|=1$ and $|a| < 1$. We denote the class of automorphisms of $\mathbb{D}$ by $\operatorname{Aut}(\mathbb{D})$. Automorphisms of $\mathbb{D}$ take $\partial\mathbb{D}$ onto $\partial\mathbb {D}$. It is known that $C_{\varphi}$ is Fredholm on the Hardy space if and only if $\varphi\in\operatorname{Aut}(\mathbb{D})$ (see [2]).

An analytic map *φ* of the disk to itself is said to have a finite angular derivative at a point *ζ* on the boundary of the disk if there exists a point *η*, also on the boundary of the disk, such that the nontangential limit as $z \rightarrow\zeta$ of the difference quotient $(\eta-\varphi(z))/(\zeta-z)$ exists as a finite complex value. We write $\varphi'(\zeta)=\angle\lim_{z \rightarrow\zeta} \frac{\eta -\varphi(z)}{\zeta-z}$.

In the second section, we investigate Fredholm and invertible weighted composition operators. In Theorem 2.7, we show that the operator $C_{\psi,\varphi}^{\ast}$ is bounded below on $H^{2}$ or $A^{2}_{\alpha}$ if and only if $C_{\psi,\varphi}$ is invertible.

In the third section, we investigate the Fredholm operators $C_{\psi _{1},\varphi_{1}}C_{\psi_{2},\varphi_{2}}^{\ast}$ and $C_{\psi _{1},\varphi_{1}}^{\ast}C_{\psi_{2},\varphi_{2}}$ on $H^{2}$ and $A^{2}_{\alpha}$.

## Bounded below operators $C_{\psi,\varphi}^{\ast}$

Let *H* be a Hilbert space. The set of all bounded operators from *H* into itself is denoted by $B(H)$. We say that an operator $A \in B(H)$ is bounded below if there is a constant $c > 0$ such that $c\| h \| \leq \|A(h)\|$ for all $h \in H$.

If *f* is defined on a set *V* and if there is a positive constant *m* such that $|f(z)| \geq m$ for all *z* in *V*, then we say that *f* is bounded away from zero on *V*. In particular, we say that *ψ* is bounded away from zero near the unit circle if there are $\delta> 0$ and $\epsilon> 0$ such that

$$\bigl\vert \psi(z) \bigr\vert > \epsilon \quad \mbox{for } \delta< |z| < 1. $$

Suppose that *T* belongs to $B(H)$. We denote the spectrum of *T*, the essential spectrum of *T*, the approximate point spectrum of *T*, and the point spectrum of *T* by $\sigma(T)$, $\sigma_{e}(T)$, $\sigma _{\mathrm{ap}}(T)$ and $\sigma_{p}(T)$, respectively. Moreover, the left essential spectrum of *T* is denoted by $\sigma_{\mathrm{le}}(T)$.

Suppose that *φ* is an analytic self-map of $\mathbb{D}$. For almost all $\zeta \in\partial\mathbb{D}$, we define $\varphi(\zeta )=\lim_{r \rightarrow1}\varphi(r\zeta)$ (the statement of the existence of this limit can be found in [17, Theorem 2.2]). If *f* is a bounded analytic function on the unit disk such that $|f(e^{i\theta})|=1$ almost everywhere, then we call *f* an inner function. We know that if *φ* is inner, then $C_{\varphi}$ is bounded below on $H^{2}$, and therefore $C_{\varphi}$ has a closed range (see [17, Theorem 3.8]).

Now we state the following simple and well-known lemma, and we frequently use it in this paper.

### Lemma 2.1

*Let* $C_{\psi,\varphi}$ *be a bounded operator on* $H^{2}$ *or* $A^{2}_{\alpha}$. *Then* $C_{\psi,\varphi}^{\ast}K_{w}=\overline{\psi(w)}K_{\varphi(w)}$ *for all* $w \in\mathbb{D}$.

In this paper, for convenience, we assume that $\gamma=1$ for $H^{2}$ and $\gamma=\alpha+2$ for $A_{\alpha}^{2}$.

### Lemma 2.2

*Suppose that* *A* *and* *B* *are two bounded operators on a Hilbert space* *H*. *If* *AB* *is a Fredholm operator*, *then* *B* *is left semi*-*Fredholm*.

### Proof

Suppose that *AB* is a Fredholm operator. Then there are a bounded operator *C* and a compact operator *K* such that $CAB=I+K$. Therefore *B* is left semi-Fredholm. □

Zhao [13] characterized Fredholm weighted composition operators on $H^{2}$. Also, Zhao [14] found necessary conditions of *φ* and *ψ* for a weighted composition operator $C_{\psi ,\varphi}$ on $A_{\alpha}^{2}$ to be Fredholm. In the following proposition, we obtain a necessary and sufficient condition for $C_{\psi,\varphi}$ to be Fredholm on $H^{2}$ and $A_{\alpha}^{2}$. Then we use it to find when $C_{\psi_{1},\varphi_{1}}^{\ast}C_{\psi _{2},\varphi_{2}}$ and $C_{\psi_{1},\varphi_{1}}C_{\psi_{2},\varphi _{2}}^{\ast}$ are Fredholm. The idea of the proof of the next proposition is different from [13] and [14].

### Proposition 2.3

*The operator* $C^{\ast}_{\psi,\varphi}$ *is left semi*-*Fredholm on* $H^{2}$ *or* $A_{\alpha}^{2}$ *if and only if* $\varphi\in\operatorname{Aut}(\mathbb{D})$ *and* $\psi\in H^{\infty}$ *is bounded away from zero near the unit circle*. *Under these conditions*, $C_{\psi,\varphi}$ *is a Fredholm operator*.

### Proof

Let $C_{\psi,\varphi}$ be Fredholm on $H^{2}$ or $A_{\alpha}^{2}$. Assume that *ψ* is not bounded away from zero near the unit circle. Then for each positive integer *n*, there is $x_{n} \in\mathbb{D}$ such that $1-1/n < |x_{n}| < 1$ and $|\psi (x_{n})| < 1/n$. Then there exist a subsequence $\{x_{n_{m}}\}$ and $\zeta\in\partial\mathbb{D}$ such that $x_{n_{m}} \rightarrow\zeta $ as $m \rightarrow\infty$. Since $\psi(x_{n_{m}})\rightarrow0$ as $m\rightarrow\infty$, by Lemma 2.1 we see that

$$\begin{aligned} \lim_{m \rightarrow\infty} \bigl\Vert C_{\psi,\varphi}^{\ast }k_{x_{n_{m}}} \bigr\Vert _{\gamma} =&\lim_{m \rightarrow\infty} \bigl\vert \psi (x_{n_{m}}) \bigr\vert \frac{\|K_{\varphi(x_{n_{m}})}\|_{\gamma}}{\| K_{x_{n_{m}}}\|_{\gamma}} \\ \leq&\limsup_{m \rightarrow\infty} \bigl\vert \psi(x_{n_{m}}) \bigr\vert \biggl(\frac {(1+|x_{n_{m}}|)(1-|x_{n_{m}}|)}{(1+|\varphi(x_{n_{m}})|)(1-|\varphi (x_{n_{m}})|)} \biggr)^{\gamma/2} \\ \leq&2^{\gamma/2}\lim_{m \rightarrow\infty} \bigl\vert \psi (x_{n_{m}}) \bigr\vert \limsup_{m \rightarrow\infty} \biggl( \frac {1-|x_{n_{m}}|}{1-|\varphi(x_{n_{m}})|} \biggr)^{\gamma/2} \\ =&0, \end{aligned}$$

where the last equality follows from the fact that $\liminf\frac {1-|\varphi(x_{n_{k}})|}{1-|x_{n_{k}}|} \neq0$ (see [17, Corollary 2.40]). Since $k_{x_{n_{m}}}$ tends to zero weakly as $m \rightarrow\infty$ (see [17, Theorem 2.17]), by [18, Theorem 2.3, p. 350], $C_{\psi,\varphi}^{\ast}$ is not left semi-Fredholm. This is a contradiction. Hence *ψ* is bounded away from zero near the unit circle. Denote the inner product on $H^{2}$ or $A_{\alpha}^{2}$ by by $\langle\cdot,\cdot\rangle_{\gamma}$, where $\gamma=1$ for $H^{2}$ and $\gamma=\alpha+2$ for $A_{\alpha }^{2}$. Define the bounded linear functional $F_{\psi}$ by $F_{\psi }(f)=\langle f,\psi\rangle_{\gamma}$ for each *f* that belongs to $H^{2}$ or $A_{\alpha}^{2}$, where $\gamma=1$ for $H^{2}$ and $\gamma =\alpha+2$ for $A_{\alpha}^{2}$. We know that, for each $\zeta\in \partial\mathbb{D}$, $K_{r\zeta}/\|K_{r\zeta}\|_{\gamma}$ tends to zero weakly as $r \rightarrow1$. Then

$$\lim_{r \rightarrow1}F_{\psi} \biggl(\frac{K_{r\zeta}}{\|K_{r\zeta }\|_{\gamma}} \biggr)= \lim_{r \rightarrow1} \biggl\langle \frac{K_{r\zeta }}{\|K_{r\zeta}\|_{\gamma}},\psi \biggr\rangle _{\gamma}=0, $$

and so $|\psi(r\zeta)|/\|K_{r\zeta}\|_{\gamma} \rightarrow0$ as $r \rightarrow1$. Now, we show that *φ* is inner. For each $\zeta\in\partial \mathbb{D}$ such that $\varphi(\zeta):=\lim_{r\rightarrow1}\varphi (r\zeta)$ exists, by Lemma 2.1 we have

$$\begin{aligned} \lim_{r \rightarrow1} \bigl\Vert C_{\psi,\varphi}^{\ast}k_{r\zeta} \bigr\Vert _{\gamma} =&\lim_{r \rightarrow1}\frac{|\psi(r\zeta)|}{\| K_{r\zeta}\|_{\gamma}} \biggl(\frac{1}{1-|\varphi(r\zeta )|^{2}} \biggr)^{\gamma/2} \\ \leq&\lim_{r \rightarrow1}\frac{|\psi(r\zeta)|}{\|K_{r\zeta}\| _{\gamma}} \biggl(\frac{1}{1-|\varphi(r\zeta)|} \biggr)^{\gamma/2}. \end{aligned}$$

Since $|\psi(r\zeta)|/\|K_{r\zeta}\|_{\gamma} \rightarrow0$ as $r \rightarrow1$, if $\varphi(\zeta) \notin\partial\mathbb{D}$, then $\lim_{r \rightarrow1}\|C_{\psi,\varphi}^{\ast}k_{r\zeta}\| _{\gamma}=0$, which is a contradiction (see [18, Theorem 2.3, p. 350]). Hence *φ* is inner. Since $C_{\psi,\varphi}^{\ast }$ is left semi-Fredholm, by Lemma 2.2, $T_{\psi}^{\ast}$ is left semi-Fredholm. Then $\operatorname{dim} \operatorname{ker} T_{\psi}^{\ast}$ is finite. It follows from Lemma 2.1 that *ψ* has only finite zeroes in $\mathbb {D}$. If *φ* is constant on $\mathbb{D}$, then $\operatorname{dim} \operatorname{ker} C_{\psi,\varphi}^{\ast}= \operatorname{dim} (\operatorname{ran} C_{\psi,\varphi})^{\bot}=\infty$, a contradiction. If $\varphi(a)=\varphi(b)$ for some $a,b \in\mathbb{D}$ with $a \neq b$, then by using the idea similar to that used in [2, Lemma] there exist infinite sets $\{a_{n}\}$ and $\{b_{n}\}$ in $\mathbb{D}$ which are disjoint and such that $\varphi(a_{n})= \varphi(b_{n})$. We can assume that $\psi(a_{n})\psi(b_{n}) \neq0$ because *ψ* has only finite zeroes in $\mathbb{D}$. By Lemma 2.1 we see that

$$C_{\psi,\varphi}^{\ast} \biggl(\frac{K_{a_{n}}}{\overline{\psi (a_{n})}}-\frac{K_{b_{n}}}{\overline{\psi(b_{n})}} \biggr)=K_{\varphi(a_{n})}-K_{\varphi(b_{n})} \equiv0. $$

Therefore, $K_{a_{n}}/\overline{\psi(a_{n})}-K_{b_{n}}/\overline {\psi(b_{n})} \in\operatorname{ker} C_{\psi,\varphi}^{\ast}$. It is not hard to see that $\{K_{a_{n}}/\overline{\psi (a_{n})}-K_{b_{n}}/\overline{\psi(b_{n})}\}$ is a linearly independent set in the kernel of $C_{\psi,\varphi}^{\ast}$, and so we have our desired contradiction. Hence *φ* must be univalent. Then [17, Corollary 3.28] implies that *φ* is an automorphism of $\mathbb{D}$. Since $C_{\psi,\varphi}C_{\varphi }^{-1}=M_{\psi}$ is a bounded multiplication operator on $H^{2}$ and $A^{2}_{\alpha}$, by [19, p. 215], $\psi\in H^{\infty}$.

Conversely, suppose that $\varphi\in\operatorname{Aut}(\mathbb{D})$ and $\psi\in H^{\infty}$ is bounded away from zero near the unit circle. Since $C_{\varphi}$ is invertible, $C_{\varphi}$ has a closed range. Since $\psi\not\equiv0$, $\operatorname{ker} T_{\psi}=(0)$. We infer that $T_{\psi}$ has a closed range by [18, Corollary 2.4, p. 352], [20, Theorem 3], and [12, Theorem 8], so by [18, Proposition 6.4, p. 208], $T_{\psi}$ is bounded below. We claim that $C_{\psi,\varphi}$ has a closed range. This can be seen as follows. Suppose that $\{h_{n}\}$ is a sequence such that $\{C_{\psi ,\varphi}(h_{n})\}$ converges to *f* as $n\rightarrow\infty$. Since $T_{\psi}$ has a closed range, $\{C_{\psi,\varphi}(h_{n})\}$ converges to $T_{\psi}g$ for some *g* as $n\rightarrow\infty$. Since $T_{\psi}$ is bounded below, there is a constant $c > 0$ such that $\|T_{\psi}(C_{\varphi}(h_{n})-g)\| \geq c\|C_{\varphi }(h_{n})-g\|$. Therefore $C_{\varphi}(h_{n}) \rightarrow g$ as $n \rightarrow\infty$. There exists *h* such that $C_{\varphi}(h)=g$ because $C_{\varphi}$ has a closed range. Hence $f=C_{\psi,\varphi }(h)$, as desired. Hence $\operatorname{ran} C_{\psi,\varphi}$ is closed and $\operatorname{ker} C_{\psi,\varphi}=(0)$. [20, Theorem 3] and [12, Theorem 10] imply that $T_{\psi}$ is Fredholm, and so $\operatorname{ker} T_{\psi}^{\ast }$ is finite dimensional. Since $\varphi\in \operatorname{Aut}(\mathbb{D})$, it is not hard to see that

$$\operatorname{ker} C_{\psi,\varphi}^{\ast}=(\operatorname{ran} C_{\psi,\varphi })^{\bot}=(\operatorname{ran} T_{\psi})^{\bot}= \operatorname{ker} T_{\psi }^{\ast}. $$

Therefore, $\operatorname{dim} \operatorname{ker} C_{\psi,\varphi}^{\ast} < \infty$, and the conclusion follows from [18, Corollary 2.4, p. 352]. □

In the next proposition, we give a necessary condition of *ψ* for an operator $C_{\psi,\varphi}^{\ast}$ to be bounded below on $H^{2}$ and $A^{2}_{\alpha}$. Then we use Proposition 2.4 to obtain all invertible weighted composition operators on $H^{2}$ and $A^{2}_{\alpha}$.

### Proposition 2.4

*Let* *ψ* *be an analytic map of* $\mathbb {D}$, *and let* *φ* *be an analytic self*-*map of* $\mathbb{D}$. *If* $C_{\psi,\varphi}^{\ast}$ *is bounded below on* $H^{2}$ *or* $A^{2}_{\alpha}$, *then* $\psi\in H^{\infty}$ *is bounded away from zero on* $\mathbb{D}$, *and* $\varphi\in\operatorname{Aut}(\mathbb{D})$.

### Proof

Let $\varphi\equiv d$ for some $d \in\mathbb{D}$. Since $C_{\psi,\varphi}^{\ast}$ is bounded below, there is a constant $c > 0$ such that $\|C_{\psi,\varphi}^{\ast} f\|_{\gamma} \geq c\|f\| _{\gamma}$ for all *f*. Then for each $w \in\mathbb{D}$, by Lemma 2.1, $\|C_{\psi,\varphi}^{\ast}K_{w}\|_{\gamma}=|\psi(w)|\|K_{d}\| _{\gamma} \geq c\|K_{w}\|_{\gamma}$. Therefore, for each $w \in\mathbb{D}$,

$$\bigl\vert \psi(w) \bigr\vert \geq\frac{c}{\|K_{d}\|_{\gamma}}\frac {1}{(1-|w|^{2})^{\gamma/2}}. $$

It is easy to see that *ψ* is bounded away from zero on $\mathbb {D}$. Now assume that *φ* is not a constant function. Suppose that *ψ* is not bounded away from zero on $\mathbb{D}$. Therefore, there exist a sequence $\{x_{n}\}$ in $\mathbb{D}$ and $a \in\overline{\mathbb{D}}$ such that $x_{n} \rightarrow a$ and $|\psi (x_{n})| \rightarrow0$ as $n \rightarrow\infty$. First, suppose that $a \in\mathbb{D}$. By Lemma 2.1 we have

$$\bigl\Vert C_{\psi,\varphi}^{\ast}k_{a} \bigr\Vert _{\gamma}= \bigl\vert \psi(a) \bigr\vert \biggl(\frac {1-|a|^{2}}{1-|\varphi(a)|^{2}} \biggr)^{\gamma/2}=0. $$

Since $C_{\psi,\varphi}^{\ast}$ is bounded below, $0 \geq c\|k_{a}\| _{\gamma}=c$, a contradiction. Now assume that $a \in\partial\mathbb {D}$. It is not hard to see that there is a subsequence $\{x_{n_{m}}\}$ such that $\{\varphi(x_{n_{m}})\}$ converges. By Lemma 2.1 we see that

1 $$ \limsup_{m \rightarrow\infty} \bigl\Vert C_{\psi,\varphi}^{\ast }k_{x_{n_{m}}} \bigr\Vert _{\gamma}=\limsup_{m\rightarrow\infty} \bigl\vert \psi (x_{n_{m}}) \bigr\vert \biggl(\frac{1-|x_{n_{m}}|^{2}}{1-|\varphi (x_{n_{m}})|^{2}} \biggr)^{\gamma/2}. $$

If $\{\varphi(x_{n_{m}})\}$ converges to a point in $\mathbb{D}$, then (1) is equal to zero. Now assume that $\{\varphi(x_{n_{m}})\}$ converges to a point in $\partial\mathbb{D}$. If *φ* has a finite angular derivative at *a*, then by the Julia-Carathéodory theorem we have

$$\limsup_{m\rightarrow\infty}\frac{1-|x_{n_{m}}|^{2}}{1-|\varphi (x_{n_{m}})|^{2}}=\frac{1}{|\varphi'(a)|}, $$

which shows that (1) is equal to zero. If *φ* does not have a finite angular derivative at *a*, then

$$\limsup_{m\rightarrow\infty}\frac{1-|x_{n_{m}}|}{1-|\varphi(x_{n_{m}})|}=0, $$

so again (1) is equal to zero. Since $C_{\psi,\varphi}^{\ast}$ is bounded below and $\|k_{x_{n_{m}}}\|_{\gamma}=1$, we have $c=0$, is a contradiction. Therefore, *ψ* is bounded away from zero on $\mathbb {D}$. Since by [18, Proposition 6.4, p. 208], $0 \notin\sigma _{\mathrm{ap}}(C_{\psi,\varphi}^{\ast})$, we have that $\lim_{r \rightarrow 1}\|C_{\psi,\varphi}^{\ast}k_{r\zeta}\|_{\gamma} \neq0$ for all $\zeta\in\partial\mathbb{D}$. We employ the idea of the proof of Proposition 2.3 to see that *φ* is a univalent inner function. Thus $\varphi\in\operatorname{Aut}(\mathbb{D})$ (see [17, Corollary 3.28]). Moreover, since $C_{\psi,\varphi}$ is a bounded operator, as we saw in the proof of Proposition 2.3, we conclude that $\psi\in H^{\infty}$, and the proposition follows. □

Bourdon [21, Theorem 3.4] obtained the following corollary; we give another proof (see also [22, Theorem 2.0.1]).

### Corollary 2.5

*Let* *ψ* *be an analytic map of* $\mathbb {D}$, *and let* *φ* *be an analytic self*-*map of* $\mathbb{D}$. *The weighted composition operator* $C_{\psi,\varphi}$ *is invertible on* $H^{2}$ *or* $A_{\alpha}^{2}$ *if and only if* $\varphi\in\operatorname{Aut}(\mathbb{D})$ *and* $\psi\in H^{\infty}$ *is bounded away from zero on* $\mathbb{D}$.

### Proof

Let $C_{\psi,\varphi}$ be invertible. Then $C_{\psi ,\varphi}^{\ast}$ is bounded below. The conclusion follows from Proposition 2.4. The reverse direction is trivial since $C_{\varphi}$ and $T_{\psi}$ are invertible. □

Note that if $C_{\psi,\varphi}$ is invertible, then $C_{\psi,\varphi }^{\ast}$ is bounded below. Hence by Proposition 2.4 and Corollary 2.5 we can see that $C_{\psi,\varphi}^{\ast}$ is bounded below if and only if $C_{\psi,\varphi}$ is invertible.

The algebra $A(\mathbb{D})$ consists of all continuous functions on the closure of $\mathbb{D}$ that are analytic on ${\mathbb{D}}$. In the next corollary, we find some Fredholm weighted composition operators that are not invertible.

### Corollary 2.6

*Suppose that* $\varphi\in\operatorname{Aut}(\mathbb {D})$ *and* $\psi\in A(\mathbb{D})$. *Assume that* $\{z \in\mathbb{D}: \psi(z)=0\}$ *is a nonempty finite set and* $\psi(z) \neq0$ *for all* $z \in\partial \mathbb{D}$. *Then* $C_{\psi,\varphi}$ *is Fredholm*, *but it is not invertible*.

### Proof

It is easy to see that *ψ* is bounded away from zero near the unit circle. Therefore the result follows from Proposition 2.3 and Corollary 2.5. □

### Theorem 2.7

*Suppose that* *ψ* *is an analytic map of* $\mathbb{D}$ *and* *φ* *is an analytic self*-*map of* $\mathbb{D}$. *The operator* $C_{\psi,\varphi}^{\ast}$ *is bounded below on* $H^{2}$ *or* $A^{2}_{\alpha}$ *if and only if* $\varphi\in\operatorname{Aut}(\mathbb {D})$ *and* $\psi\in H^{\infty}$ *is bounded away from zero on* $\mathbb {D}$.

## The operators $C_{\psi_{1},\varphi_{1}}C_{\psi_{2},\varphi _{2}}^{\ast}$ and $C_{\psi_{1},\varphi_{1}}^{\ast}C_{\psi _{2},\varphi_{2}}$

In this section, we find all Fredholm operators $C_{\psi_{1},\varphi _{1}}C_{\psi_{2},\varphi_{2}}^{\ast}$ and $C_{\psi_{1},\varphi _{1}}^{\ast}C_{\psi_{2},\varphi_{2}}$.

A linear-fractional self-map of $\mathbb{D}$ is a mapping of the form $\varphi(z)=(az+b)/(cz+d)$ with $ad-bc \neq0$ such that $\varphi (\mathbb{D}) \subseteq\mathbb{D}$. We denote the set of those maps by $\operatorname{LFT}(\mathbb{D})$. Suppose $\varphi(z)=(az+b)/(cz+d)$. It is well known that the adjoint of $C_{\varphi}$ acting on $H^{2}$ and $A^{2}_{\alpha}$ is given by $C_{\varphi}^{\ast}=T_{g}C_{\sigma}T_{h}^{\ast}$, where $\sigma(z)=({\overline{a}z-\overline{c}})/({-\overline {b}z+\overline{d}})$ is a self-map of $\mathbb{D}$, $g(z)=(-\overline{b}z+\overline {d})^{-\gamma}$, and $h(z)=(cz+d)^{\gamma}$. Note that *g* and *h* are in $H^{\infty}$ ([17, Theorem 9.2]). If $\varphi(\zeta)=\eta$ for $\zeta,\eta\in\partial\mathbb{D}$, then $\sigma(\eta)=\zeta$. We know that *φ* is an automorphism if and only if *σ* is, and in this case, $\sigma=\varphi^{-1}$. The map *σ* is called the Krein adjoint of *φ*. We denote by $F(\varphi)$ the set of all points in $\partial\mathbb{D}$ at which *φ* has a finite angular derivative.

### Example 3.1

Suppose that $\varphi\in\operatorname{LFT}(\mathbb{D})$ is not an automorphism of $\mathbb{D}$. Assume that $\psi\in H^{\infty}$ is continuously extendable to $\mathbb{D} \cup F(\varphi )$. Assume that $C_{\psi,\varphi}C_{\psi,\varphi}^{\ast}$ is considered as an operator on $H^{2}$ or $A_{\alpha}^{2}$. Since *φ* is not an automorphism of $\mathbb{D}$, $\overline {\varphi(\mathbb{D})} \subseteq\mathbb{D}$ or there is only one point $\zeta\in\partial\mathbb{D}$ such that $\varphi(\zeta) \in \partial\mathbb{D}$. If $\overline{\varphi(\mathbb{D})} \subseteq \mathbb{D}$, then by [17, p. 129], $C_{\varphi}$ is compact. It is easy to see that $C_{\psi,\varphi}C_{\psi,\varphi}^{\ast}$ is a compact operator. Since compact operators are not Fredholm, we can see that $C_{\psi,\varphi}C_{\psi,\varphi}^{\ast}$ is not Fredholm.

In the other case, assume that $F(\varphi)=\{\zeta\}$. Because for each $w \in\partial\mathbb{D}$ such that $w \neq\zeta$, $\sigma (\varphi(w)) \notin\partial\mathbb{D}$, we obtain $\sigma \circ \varphi\notin\operatorname{Aut}(\mathbb{D})$. Since $C_{\sigma\circ \varphi}$ is not Fredholm (see e.g. [2] and [16]), $0 \in \sigma_{e}(C_{\sigma\circ\varphi})$. By [23, Corollary 2.2] and [4, Proposition 2.3] there is a compact operator *K* such that

$$C_{\psi,\varphi}C_{\psi,\varphi}^{\ast}= \bigl\vert \psi(\zeta ) \bigr\vert ^{2}C_{\varphi}C_{\varphi}^{\ast}+K. $$

Also, [23, Theorem 3.1], [23, Proposition 3.6], and [24, Theorem 3.2] imply that there is a compact operator $K'$ such that

2 $$ C_{\psi,\varphi}C_{\psi,\varphi}^{\ast}= \bigl\vert \psi(\zeta ) \bigr\vert ^{2} \bigl\vert \varphi'(\zeta) \bigr\vert ^{-\gamma}C_{\sigma\circ\varphi}+K'. $$

From the fact that $0 \in\sigma_{e}(C_{\sigma\circ\varphi})$ and equation (2) we can infer that $0 \in\sigma_{e}(C_{\psi,\varphi }C_{\psi,\varphi}^{\ast})$. Then $C_{\psi,\varphi}C_{\psi,\varphi }^{\ast}$ is not Fredholm.

By the preceding example it seems natural to conjecture that if $C_{\psi,\varphi}C_{\psi,\varphi}^{\ast}$ is Fredholm, then $\varphi\in\operatorname{Aut}(\mathbb{D})$. We will prove our conjecture in Theorem 3.2 and show that if $C_{\psi_{1},\varphi_{1}}C_{\psi _{2},\varphi_{2}}^{\ast}$ is Fredholm on $H^{2}$ or $A_{\alpha }^{2}$, then $C_{\psi_{1},\varphi_{1}}$ and $C_{\psi_{2},\varphi _{2}}$ are Fredholm.

### Theorem 3.2

*The operator* $C_{\psi_{1},\varphi_{1}}C_{\psi _{2},\varphi_{2}}^{\ast}$ *is Fredholm on* $H^{2}$ *or* $A_{\alpha}^{2}$ *if and only if* $\varphi_{1},\varphi_{2} \in\operatorname{Aut}(\mathbb {D})$, $\psi_{1},\psi_{2} \in H^{\infty}$, *and* $\psi_{1}$ *and* $\psi _{2}$ *are bounded away from zero near the unit circle*.

### Proof

Let $C_{\psi_{1},\varphi_{1}}C_{\psi_{2},\varphi _{2}}^{\ast}$ be Fredholm. Therefore $C_{\psi_{2},\varphi_{2}}^{\ast }$ is left semi-Fredholm. By Proposition 2.3 we see that $\varphi_{2} \in\operatorname{Aut}(\mathbb{D})$ and $\psi_{2} \in H^{\infty}$ is bounded away from zero near the unit circle. Since $C_{\psi _{2},\varphi_{2}}C_{\psi_{1},\varphi_{1}}^{\ast}$ is Fredholm, again we can see that $\varphi_{1}$ is an automorphism of $\mathbb {D}$ and $\psi_{1} \in H^{\infty}$ is bounded away from zero near the unit circle.

Conversely, let $\varphi_{1},\varphi_{2} \in\operatorname{Aut}(\mathbb {D})$ and $\psi_{1},\psi_{2} \in H^{\infty}$ be bounded away from zero near the unit circle. By Proposition 2.3, $C_{\psi_{1},\varphi _{1}}$ and $C_{\psi_{2},\varphi_{2}}^{\ast}$ are Fredholm, so the result follows. □

In the following theorem for functions $\psi_{1},\psi_{2} \in A(\mathbb{D})$, we find all Fredholm operators $C_{\psi_{1},\varphi _{1}}^{\ast}C_{\psi_{2},\varphi_{2}}$ when $\varphi_{1}$ and $\varphi_{2}$ are univalent self-maps of $\mathbb{D}$.

### Theorem 3.3

*Suppose that* $\psi_{1},\psi_{2} \in A(\mathbb{D})$. *Let* $\varphi_{1}$ *and* $\varphi_{2}$ *be univalent self*-*maps of* $\mathbb{D}$. *The operator* $C_{\psi_{1},\varphi _{1}}^{\ast}C_{\psi_{2},\varphi_{2}}$ *is Fredholm on* $H^{2}$ *or* $A_{\alpha}^{2}$ *if and only if* $C_{\psi_{1},\varphi_{1}}$ *and* $C_{\psi_{2},\varphi_{2}}$ *are Fredholm on* $H^{2}$ *or* $A_{\alpha }^{2}$, *respectively*.

### Proof

Let $C_{\psi_{1},\varphi_{1}}^{\ast}C_{\psi _{2},\varphi_{2}}$ be Fredholm on $H^{2}$ or $A_{\alpha}^{2}$. Then $C_{\psi_{2},\varphi_{2}}^{\ast}C_{\psi_{1},\varphi_{1}}$ is also Fredholm. It is easy to see that $C_{\varphi_{2}}$ and $C_{\varphi _{1}}$ are left semi-Fredholm. Therefore, $0 \notin\sigma _{\mathrm{le}}(C_{\varphi_{1}})$ and $0 \notin\sigma_{\mathrm{le}}(C_{\varphi _{2}})$. Since $\operatorname{dim} \operatorname{ker} C_{\psi_{1},\varphi _{1}}^{\ast}C_{\psi_{2},\varphi_{2}} < \infty$ and $\operatorname{dim} \operatorname{ker} C_{\psi_{2},\varphi_{2}}^{\ast}C_{\psi _{1},\varphi_{1}} < \infty$, $\psi_{1} \not\equiv0$, $\psi_{2} \not\equiv0$, and $\varphi _{1}$ and $\varphi_{2}$ are not constant functions. By the open mapping theorem we know that $0 \notin\sigma_{p}(C_{\varphi_{1}})$ and $0 \notin\sigma_{p}(C_{\varphi_{2}})$. Now [18, Proposition 4.4, p. 359] implies that $0 \notin\sigma_{\mathrm{ap}}(C_{\varphi _{1}})$ and $0 \notin\sigma_{\mathrm{ap}}(C_{\varphi_{2}})$. Hence by [18, Proposition 6.4, p. 208], $\operatorname{ran} C_{\varphi_{1}}$ and $\operatorname{ran} C_{\varphi_{2}}$ are closed. By [1, Theorem 5.1], $\varphi_{1},\varphi_{2} \in\operatorname{Aut}(\mathbb{D})$. Since $C_{\varphi_{1}}^{\ast}$ and $C_{\varphi_{2}}$ are invertible, $(C_{\varphi_{1}}^{\ast})^{-1}$ and $C_{\varphi_{2}}^{-1}$ are Fredholm. Then $T_{\psi_{1}}^{\ast}T_{\psi_{2}}$ is Fredholm, and so $0 \notin\sigma_{e}(T_{\overline{\psi_{1}}\psi_{2}})$. We get from [25] and [20, Theorem 2] that $\psi_{1}$ and $\psi _{2}$ never vanish on $\partial\mathbb{D}$. Since $\psi_{1} \not \equiv0$ and $\psi_{2} \not\equiv0$, $\psi_{1}$ and $\psi_{2}$ have only finite zeroes on $\mathbb{D}$. This implies that there is $r < 1$ such that for each *w* with $r < |w| < 1$, $\psi_{1}(w) \neq0$ and $\psi_{2}(w) \neq0$. Therefore, $\psi_{1}$ and $\psi_{2}$ are bounded away from zero near the unit circle. Therefore, by Proposition 2.3, $C_{\psi_{1},\varphi_{1}}$ and $C_{\psi_{2},\varphi_{2}}$ are Fredholm on $H^{2}$ or $A_{\alpha}^{2}$. The reverse implication follows from the fact stated before Theorem 3.2. □

## References

[1] Akeroyd JR, Ghatage PG (2008). Closed-range composition operators on $A^{2}$. Ill. J. Math..

[2] Bourdon PS (1990). Fredholm multiplication and composition operators on the Hardy space. Integral Equ. Oper. Theory.

[3] Cowen CC (1988). Linear fractional composition operators on $H^{2}$. Integral Equ. Oper. Theory.

[4] Fatehi M, Haji Shaabani M (2015). Some essentially normal weighted composition operators on the weighted Bergman spaces. Complex Var. Elliptic Equ..

[5] Hurst P (1997). Relating composition operators on different weighted Hardy spaces. Arch. Math. (Basel).

[6] Li S, Stević S (2008). Riemann-Stieltjes operators between different weighted Bergman spaces. Bull. Belg. Math. Soc. Simon Stevin.

[7] Stević S (2007). Continuity with respect to symbols of composition operators on the weighted Bergman space. Taiwan. J. Math..

[8] Stević S (2012). Weighted iterated radial operators between different weighted Bergman spaces on the unit ball. Appl. Math. Comput..

[9] Stević S, Sharma AK, Bhat A (2011). Products of multiplication composition and differentiation operators on weighted Bergman spaces. Appl. Math. Comput..

[10] Stević S, Sharma AK, Bhat A (2011). Essential norm of products of multiplication composition and differentiation operators on weighted Bergman spaces. Appl. Math. Comput..

[11] Stević S, Ueki S-I (2010). Weighted composition operators from the weighted Bergman space to the weighted Hardy space on the unit ball. Appl. Math. Comput..

[12] Vukotić D (2003). Analytic Toeplitz operators on the Hardy space $H^{p}$: a survey. Bull. Belg. Math. Soc. Simon Stevin.

[13] Zhao L (2013). Fredholm weighted composition operator on Hardy space. J. Math. Res. Appl..

[14] Zhao L (2013). Fredholm weighted composition operator on weighted Hardy space. J. Funct. Spaces Appl..

[15] Cima JA, Thomson J, Wogen W (1974). On some properties of composition operators. Indiana Univ. Math. J..

[16] MacCluer BD (1997). Fredholm composition operators. Proc. Am. Math. Soc..

[17] Cowen CC, MacCluer BD (1995). Composition Operators on Spaces of Analytic Functions.

[18] Conway JB (1990). A Course in Functional Analysis.

[19] Halmos P (1974). A Hilbert Space Problem Book.

[20] Perälä A, Virtanen JA (2011). A note on the Fredholm properties of Toeplitz operators on weighted Bergman spaces with matrix-valued symbols. Oper. Matrices.

[21] Bourdon PS (2014). Invertible weighted composition operators. Proc. Am. Math. Soc..

[22] Gunatillake G (2011). Invertible weighted composition operators. J. Funct. Anal..

[23] Kriete TL, MacCluer BD, Moorhouse JL (2007). Toeplitz-composition $C^{\ast}$-algebras. J. Oper. Theory.

[24] MacCluer BD, Narayan SK, Weir RJ (2013). Commutators of composition operators with adjoints of composition operators on weighted Bergman spaces. Complex Var. Elliptic Equ..

[25] Coburn LA (1969). The $C^{\ast}$-algebra generated by an isometry. II. Trans. Am. Math. Soc..

